# Assessment of confidence in medical writing: Development and validation of the first trustworthy measurement tool

**DOI:** 10.1371/journal.pone.0302299

**Published:** 2024-04-18

**Authors:** Behrooz Astaneh, Hadi Raeisi Shahraki, Vala Astaneh, Gordon Guyatt

**Affiliations:** 1 Faculty of Health Sciences, Department of Health Research Methods, Evidence and Impact, McMaster University, Hamilton, Ontario, Canada; 2 Department of Epidemiology and Biostatistics, School of Health, Shahrekord University of Medical Sciences, Shahrekord, Iran; 3 Faculty of Kinesiology and Health Sciences, York University, Toronto, Ontario, Canada; 4 Department of Health Research Methods, Evidence and Impact and Department of Medicine, McMaster University, Hamilton, Ontario, Canada; Qatar University College of Nursing, QATAR

## Abstract

**Background:**

The popularity of medical writing workshops highlights the need for a standard measurement tool to assess the impact of such workshops on participants’ confidence in: 1- writing a standard article and 2- using optimal English language. Because such an instrument is not yet available, we undertook this study to devise and evaluate the first measurement tool to assess such confidence.

**Method:**

We created an item pool of 50 items by searching Medline, Embase, and Clarivate Analytics to find related articles, using our prior experience, and approaching the key informants. We revised and edited the item pool, and redundant ones were excluded. Finally, the 36-item tool comprised two domains. We tested it in a group of workshop applicants for internal consistency and temporal reliability using Cronbach’s α and Pearson correlations and for content and convergent validity using the content validity index and Pearson correlations.

**Results:**

The participants had a mean age of 40.3 years, a female predominance (74.3%), and a majority of faculty members (51.4%). The internal consistency showed high reliability (> 0.95). Test-retest reliability showed very high correlations (r = 0.93). The CVI for domain 1 was 0.78, for domain 2 was 0.73, and for the entire instrument was 0.75.

**Conclusion:**

This unique, reliable, and valid measurement tool could accurately measure the level of confidence in writing a standard medical article and in using the appropriate English language for this purpose.

## Introduction

Effective medical writing and success in publishing the results of studies in peer-reviewed journals are two crucial steps in disseminating the findings to international audiences [1,2]. Without publishing the results, the whole process of conception, design, data gathering, analyses, and interpretations of data would be largely wasted. Success in medical writing and publishing is considered a measure of personal credibility and a key criterion for career development and academic promotion [3,4]. Being successful in publishing in peer-reviewed medical journals requires capabilities such as knowing how to write and structure different sections of one’s paper based on the recommendations of international organizations like the International Committee of Medical Journal Editors (ICMJE) [5]; using the optimal English language for academic medical writing, and knowledge of the criteria and ethics of issues such as authorship.

Careful selection of words, use of the active voice, short paragraphs, and transparent connections between paragraphs are among the recommendations for optimal use of English in scientific writing [6]. Those whose first language is not English face particular challenges [7,8]. Lack of knowledge of publication ethics is the main reason why people commit ethical misconduct [9,10]. These are challenges that all researchers in various fields of health research must overcome [11].

Teaching these issues could ideally occur in graduate health sciences courses. In general, however, research-intensive graduate programs in health sciences do not emphasize writing and publishing challenges. Thus, extracurricular teaching sessions and workshops have a potentially valuable role and are continuously being held in different academic medical centers with different educational teaching plans and different target audiences [12,13]. However, the impact of such workshops in increasing the confidence of participants in medical writing skills has not been evaluated, mostly because confidence in this subjective assessment requires a reliable and valid instrument. Confidence plays an important role in authors’ success: self-doubt impairs productivity and morale, while confidence facilitates productivity and enhances morale. Moreover, confidence will be positively associated with competence [14].

Considering the popularity of medical writing workshops and educational sessions, a need exists for a standard measurement tool to assess participants’ confidence in: 1- writing different parts of a standard article based on international guidelines and 2- knowing the appropriate style and optimal academic English language required for publishing the articles in peer-reviewed medical journals. If confidence is measured using a standard instrument, it can constitute a criterion for assessing the success of writing and publishing workshops. Educators can potentially use the pre-scores obtained in each domain and item of the tool as a guide for preparing related materials for teaching based on the needs and requirements of the participants. Comparisons of scores before and after the workshops, or in randomized trials of workshops, can inform their success.

Because an instrument measuring confidence in medical writing is not yet available, we undertook a study to devise and evaluate the first measurement tool to assess authors’ confidence in writing different parts of a standard medical article and in using the optimal English language required for the preparation of such articles.

## Methods

### Item & measurement development

To find if there is any similar instrument that could be modified and used for the purpose of assessing confidence in medical writing and using the related English language, we conducted a comprehensive literature search. Failing to find a similar tool, we created an item pool for devising a new tool. To do so, we conducted a comprehensive search for articles addressing medical writing and any possible assessments done during writing workshops. Using keywords that included “medical writing workshop”, “confidence”, “medical writing”, and “journalology”, we searched Medline, Embase, and Clarivate Analytics to find related articles.

We also used our own experience in conducting many such workshops in which we have interacted with participants to identify areas in which they feel more or less confident. These observations provided ideas regarding the categories of problems that authors may face while writing their articles and how such problems might be addressed.

We also sought comments from medical journal editors through related forums, including the World Association of Medical Editors (WAME) and Eastern Mediterranean Association of Medical Editors (EMAME), which led to snowballing through which we met and interacted with other informants of the field who then provided help in choosing possible items for inclusion in the tool. This led to interactions with three university professors who had acted as the supervisors of students of graduate programs (MSc, Ph.D.) and who had many years of experience in facilitating medical writing workshops. These interactions helped us reach a “saturation” of topics and themes that should be included in the tool.

On the basis of our literature review, our prior experience, and the key informants, we constructed an item pool containing 50 items. In wording the items, we avoided colloquial words, overly technical language, and ambiguous wording. We revised and edited the item pool several times, and the best items were selected and redundant ones excluded. Finally, we arrived at a 37-item tool comprising two domains.

A panel of five experts, including two Editors-in-Chief of medical journals for >20 years, one workshop presenter and National Board examiner, one university lecturer specializing in teaching medical writing, and one graduate of the Master’s program of medical journalology, rated the items. S1 File provides a full description of the panel members’ credentials. Based on the feedback of the panel, an item that was related to “confidence in writing clinical implication of the findings” was removed because the panel thought this item might not be applied to all types of medical articles.

### Measures

We prepared a Google form to present the final items in domains of “confidence in choosing the appropriate contents for different parts of a standard medical article” based on the international guidelines (18 items), and “confidence in the use of appropriate academic English language required for publishing articles in peer-reviewed medical journals” (18 items). The response option for each item was a 5-point Likert scale ranging from 1 (no confidence) to 5 (extremely confident). The instrument typically takes less than 8 minutes to complete.

### Instrument testing

In order to test the psychometric properties of the instrument, we approached the applicants of our previous workshops whose email addresses we had collected for future workshops in March 2021. We contacted them and explained the benefits of devising our instrument and our aims in testing its measurement properties and asked for their consent to be included in testing. The agreement to complete the instrument implied informed consent while we kept their data confidential. We offered them an incentive of having priority for future workshops and emailed them the related Google form link and also sent the link through WhatsApp, asking them to fill out the form in a maximum of 2 days. We followed up with them after 1–2 weeks and requested they once again complete the instrument. The Google form also contained questions related to the demographic characteristics of the participants.

### Statistical analysis

Descriptive statistics such as mean, SD and range as well as and frequency (%) were applied to describe the quantitative and qualitative variables respectively.

The reliability of the instrument was checked using Cronbach’s α and Pearson correlations.

To check the content validity, we first calculated the content validity ratio (CVR) for each item based on the ratings of the expert panel using the formula: CVR = [ne-N/2] ÷ N/2, where ne = number of experts who rated an item as extremely relevant, and N = total number of experts [15]. The expert panel was asked to rate each item on a 4-point scale, and scores 3 and 4 for each item were considered extremely relevant. The mean of all items was considered as the content validity index (CVI). CVI was calculated for each domain separately and also for the whole instrument.

To assess convergent validity, we calculated the Pearson correlation coefficient between the total score for domain 1 and the total score for domain 2.

To address the suitable selections of the domains and the entire construct, we conducted a quasi-confirmatory factor analysis. The unrotated matrix and rotated matrix were evaluated, and a scree plot was prepared to assess for a clear elbow and for possible factors with Eigenvalue>1 [16].

Statistical analyses were done using SPSS software version 25.

## Results

All 35 people whom we approached for the pilot testing agreed to participate in the testing and filled out the form in 1–2 days, with a response rate of 100%. Of these, 29 of 35 responded to the re-test, an 83% response rate.

Table 1 presents the demographic characteristics of the participants, with a mean age of 40.3 years, a female predominance, and a majority of faculty members.

**Table 1:** Demographic data of the participants (*N* = 35).

**Age (year)**	**Mean**	**40.26**
	SD	7.62
	Range	27–56
**Sex (*No*. %)**	Male	*9* (25.7)
	Female	*26* (74.3)
**Level of education (*No*. %)**	BA	*1* (2.9)
	MSc	*11* (31.4)
	PhD	*13* (37.1)
	MD	*7* (20)
	DDS	*3* (8.6)
**Academic career (*No*. %)**	Student	*7* (20)
	Faculty member	*18* (51.4)
	Independent researcher	*10* (28.6)
Number of previous publications (*No*. %)	≤5	*14* (40)
	6–10	*7* (20)
	≥10	*14* (40)

Abbreviations: BA: Bachelor of Arts, MSC: Master of Science, PhD: Doctor of Philosophy, MD: Doctor of Medicine, DDS: Doctor of Dental Surgery.

### Reliability

Table 2 shows the internal consistency for each domain and for the total tool showing high reliability. Temporal stability was checked using test-retest reliability. Table 3 shows the related test for each domain and for the total tool, showing very high correlations.

**Table 2:** Internal consistency for each domain and for the total tool for the two time periods.

Internal Consistency assessed by Crobach’s α		
**Time 1**	Domain 1	0.96
	Domain 2	0.97
	Total	0.98
**Time 2**	Domain 1	0.97
	Domain 2	0.97
	Total	0.98

**Table 3:** The results of test re-test reliability analysis for both domains in the two different periods.

Test Re-test Reliability		
Time 1	Time 2	r
**Domain 1**	Domain 1	r = 0.84
**Domain 2**	Domain 2	r = 0.95
**Total**	Total	r = 0.93

### Content validity

The CVI for domain 1 was 0.78, for domain 2 was 0.73, and for the entire instrument was 0.75. S2 File shows the CVR of all the individual items in both domains.

The correlation between the total score for domain 1 and the total score for domain 2 was strongly positive and significant (r = 0.79, P < 0.001).

**Fig 1:**
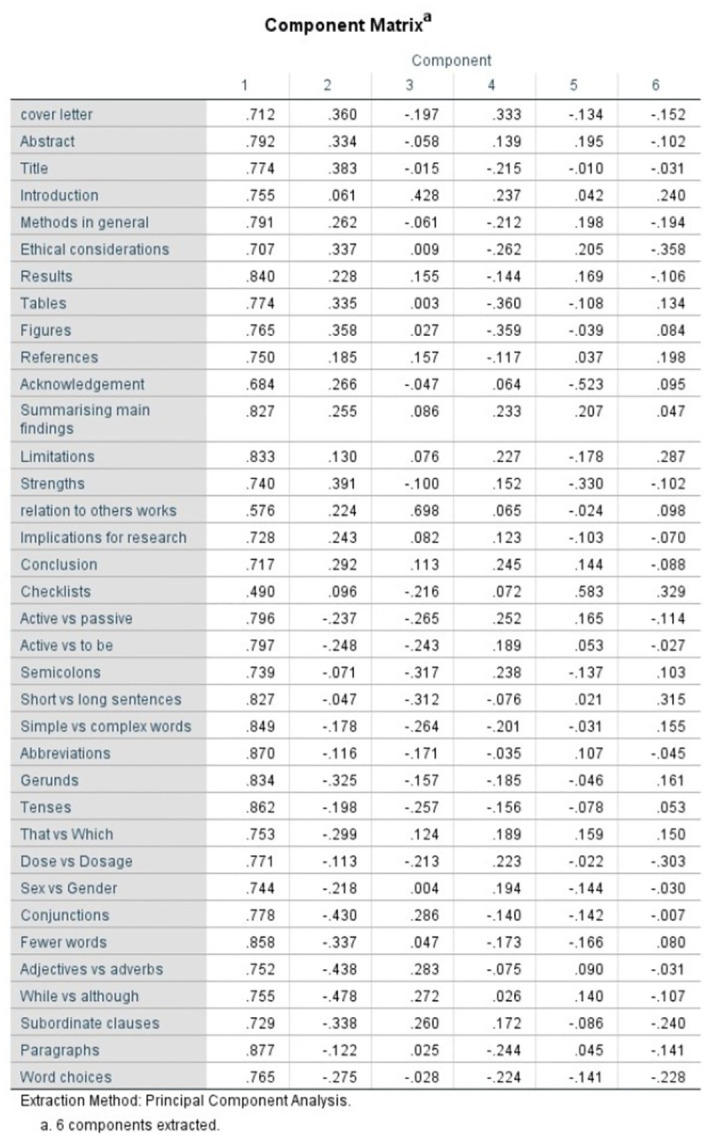
The unrotated matrix showing unidimentionality.

**Fig 2:**
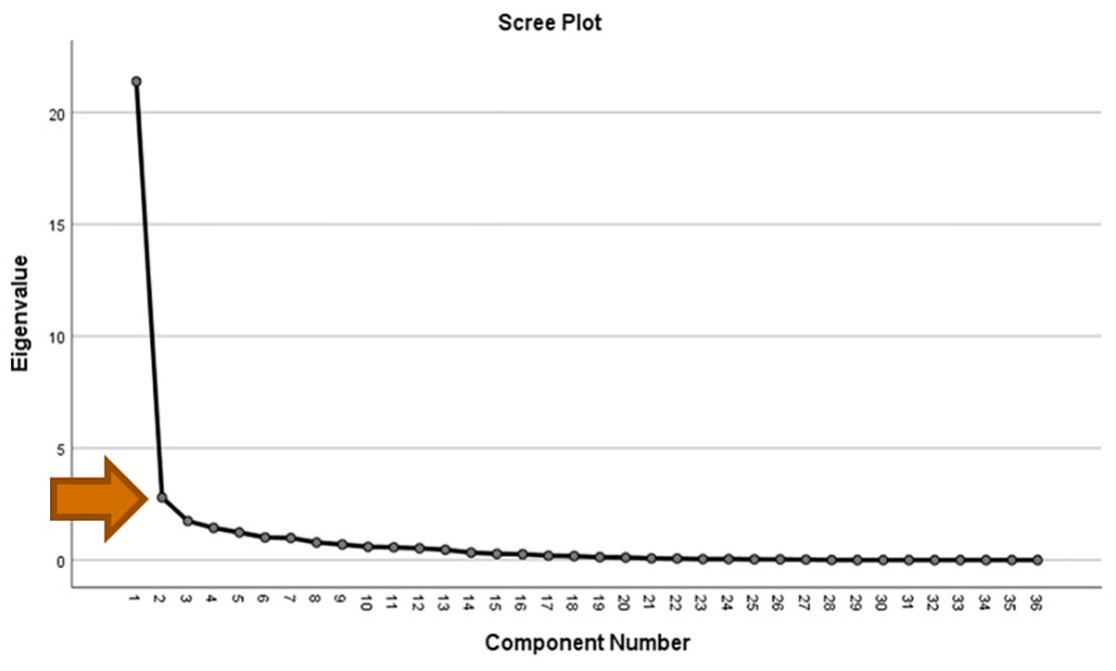
Scree plot showing an elbow at factor 2.

In quasi-confirmatory factor analysis, the unrotated matrix showed that 33 out of the 36 items loaded onto the first factor at a loading > 0.7, confirming the unidimensionality (Fig 1). Having considered factor loading > 0.4 as acceptable, the rotated matrix showed two clear factors, consistent with our conceptualization. Fig 2 shows the scree plot with a clear elbow at factor 2, also confirming our conceptualization.

The full measurement tool is attached as S3 File.

S4 File shows the rankings of the items by the expert panel.

## Discussion

We devised an instrument with two domains to assess the confidence in writing a standard medical article and in using optimal English language to write such articles. Each domain has 18 items. Domain 1 evaluates the confidence in writing different parts of a standard medical article, from writing the cover letter to writing the conclusion of the article. Domain 2 evaluates confidence in using active verbs, short sentences, simple words, gerunds, conjunctions etc. With an internal consistency of about 0.97 and test-retest reliability of 0.926, we found the instrument to be highly reliable. In terms of the interpretation of the results of the internal consistency findings, subsequent users of the instrument can be confident that it captures a single construct of confidence in academic writing ability and that all items contribute to that construct. And in terms of the interpretation of the results of the test-retest reliability analysis, subsequent users can be confident that scores will not change appreciably in the absence of true change in respondents’ confidence in their academic writing skills.

The CVI of 0.75 and convergent validity (the correlation between the two domains) of 0.79 showed the tool has acceptable validity. As shown in Fig 1, factor analysis confirmed unidimensionality, which further confirms that the items measure the same property. This adds to the construct validity of the scale. Unidimensionality also has an influence on the reliability of the test [17]. All these confirm the suitable selections of the domains and the entire instrument.

### Strengths and limitations

This is the first measurement instrument with demonstrated reliability and validity for use in all medical writing workshops as well as other similar educational meetings to assess how participants feel competent in writing medical articles using the optimal English language. Recruiting experts that allowed quantitative assessment of content validity is another strength of this study. Assessment of both internal consistency and test-retest reliability and addressing validity, including convergent validity, are other strengths. And finally, using factor analysis to confirm the suitable selections of the domains and the entire construct is the other important strength of our study. However, this study has its own limitations. We could not check for criterion and discriminative validity because there was no similar tool with which we could compare. Future studies in this regard will have the potential to address this limitation by comparing their tools with ours as the only available tool.

We might have used a larger sample size. We made considerable efforts to secure the sample size we did, and a larger sample size proved unfeasible.

### Relation to prior work

Although many researchers have tried to assess the impact of educational sessions on medical writing, most have used a qualitative approach to assess the participants’ feelings toward the session or used surveys to assess writing apprehensions [18,19]. Other studies that reported the use of quantitative instruments lack the required information regarding their psychometric properties [20]. Cargail and colleagues evaluated the confidence of the participants in their writing program psychometric properties of the used instrument was provided [21]. In a mixed method study to evaluate the outcome of writing seminars, Gardners and co-workers performed some interviews with the participants and also performed a pre-post study to assess the writing apprehension using the Writing Apprehension Test (WAT) and writing attitude using Writing Attitude Questionnaire (WAQ). Again, no info about the psychometric properties of the instruments in the new population under study was provided [19].

### Implications for practice and research

We sought expert panel feedback to calculate the CVI. The experts carefully reviewed the items, and their feedback consistently indicated that alteration was necessary for only one item. This particular item inquired about participants’ confidence in writing the clinical implications of their studies. Upon evaluation by our expert panel, they believed that including such an item might limit the tool’s applicability to only clinical studies. Recognizing the potential benefit of a broader scope, which could enhance the tool’s usability across various contexts, we decided to delete that specific item. This adjustment aims to contribute to the overall impact and versatility of the tool.

Although speaking and sharing ideas with experts and performing required modifications in items could be considered as a means of content validity, to evaluate the validity of our measurement tool, we also calculated the content validity index. Content validity is demonstrated when the items within the instrument encompass the entire spectrum of the attribute being investigated [15]. CVI is the most widely used index in the quantitative evaluation of the validity of a measurement tool [22]. It has various advantages over alternative indexes, such as ease of computation, understandability, and provision of both item and scale information [23]. With a total CVI = 0.75, our tool showed acceptable content validity.

To check for internal consistency, we used Cronbach’s α. Internal consistency shows how well different items of a tool fit together conceptually [15]. In this project, the Cronbach’s α for each domain and for the total tool was more than 0.90, which is in accordance with some authorities who believe that Cronbach’s α for such instruments should be 0.90 at minimum [24]. This high internal consistency can reflect the relatively large number of items as some researchers believe that the higher the number of items in a tool, the higher the coefficient α values [25], and for tools with more than 15 items, there will be a very high coefficient α [26]. Our tool had 36 items in total and 18 items in each domain. It should be considered that reliability is a fraction of signal over noise [27]. The signal here is between-individuals variance, which, by its increase, the numerator will increase, and then the reliability will increase too. However, the variability between individuals is different from the variability within individuals [28]. In other words, if, for example, two items of confidence in writing the “Method” and confidence in writing the “Results” are too correlated, this can be interpreted as anybody who is confident in writing the “Results” is also confident in writing the “Method”; however, it is possible that in real-world and after educational intervention, confidence in writing the “Results” increases in one participant while confidence in writing the “Methods” increases in another participant, which shows that within-individual changes after the intervention may be different. The analysis of test-retest correlation in our study also showed that correlations in both domains are well above the threshold for acceptability.

## Conclusion

Translating subjective feelings of confidence in medical writing through a reliable, objective measure has always been required. This unique, reliable, and valid measurement tool could accurately measure the level of confidence in writing a standard medical article and also in using the appropriate English language for this purpose. Future projects can focus on assessing the confidence of participants in publishing issues such as journal selection, scientometric issues, and how to prevent ethical misconduct.

## Supporting information

S1 File Expert panel profile. (DOCX)

S2 File CVR of all items in domain 1 and domain 2. (DOCX)

S3 File Final tool for confidence in medical writing. (PDF)

S4 File Expert rankings of the items in both domains. (DOCX)
