# Supplementary material for: Assessment of confidence in medical writing: Development and validation of the first trustworthy measurement tool
Source: PLoS One. 2024 Apr 18;19(4):e0302299. doi: 10.1371/journal.pone.0302299 (PMC11025726; doi:10.1371/journal.pone.0302299)
Supplement: S1 File — (DOCX) [file pone.0302299.s001.docx]

**Expert panel profile**

Expert 1: He is a full professor of dermatology with a specific interest in scientific writing and medical journalology. He has 20 years of experience as an Editor-in-Chief of a medical journal in the field of cancer and the same period background in medical writing and publishing workshops. He is the President of the Eastern Mediterranean Association of Medical Editors (EMAME) and one of the past directors of the World Association of Medical Editors.

Expert 2: He is a full professor of parasitology with an interest and background in running medical writing and publishing workshops. He has 25 years of experience as the Editor-in-Chief of a general health journal. He is one of the past vice presidents of EMAME.

Expert 3: She is a university lecturer in medical journalology and scientific writing for more than 10 years. She has also been the senior editor of a reputable medical journal for about 10 years.

Expert 4: She is a full professor of Immunology with a long-standing background in lecturing scientific writing and medical publishing. She is an examiner in the National Board of medical journalology for more than 10 years.

Expert 5: She is a graduate in medical journalology with excellence. She has been active in organizing and running medical writing and publishing journals during the last 5 years.
