# Supplementary figures and images for: Assessment of confidence in medical writing: Development and validation of the first trustworthy measurement tool
Source: PLoS One. 2024 Apr 18;19(4):e0302299. doi: 10.1371/journal.pone.0302299 (PMC11025726; doi:10.1371/journal.pone.0302299)

**CVR of all items in domain 1 and domain 2**

**
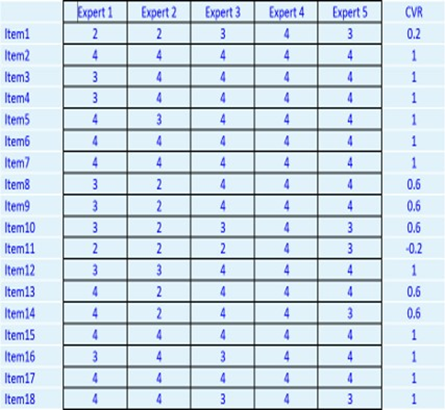

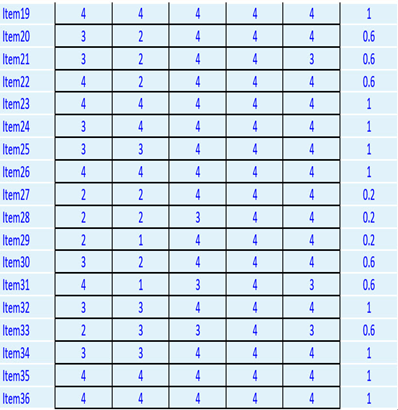
**

Supplement: S2 File — (DOCX) [file pone.0302299.s002.docx]
