# Supplementary material for: Assessment of confidence in medical writing: Development and validation of the first trustworthy measurement tool
Source: PLoS One. 2024 Apr 18;19(4):e0302299. doi: 10.1371/journal.pone.0302299 (PMC11025726; doi:10.1371/journal.pone.0302299)
Supplement: S3 File — (PDF) [file pone.0302299.s003.pdf]

“Confidence” in writing for medical journals

Consider that you have performed a study and are going to prepare an article for submission to a medical journal for peer review. We would like to understand how confident you feel in preparing the article for submission, and have some questions regarding your confidence. By confident, we are referring to judging your ability to ultimately submit a top-quality paper. In answering each of the questions, please use the following 1 to 5 scale:
1 – No confidence
2 – Low confidence
3 – Moderate confidence
4 – High confidence
5 – Very high confidence

* Required

1. Email address *

____

2. Age *

____

3. Sex *

Mark only one oval.

- ( ) male
- ( ) Female
- ( ) Prefer not to say
- ( ) Other: _______________

4. Highest level of education *

Mark only one oval.

- MSc
- PhD
- MD
- Other: ____________________

5. Your academic career *

Mark only one oval.

- Student
- Faculty member
- Independent researcher
- Other: ____________________

Domain 1

“Confidence” in choosing the appropriate contents for different parts of a standard medical article based on the international guidelines.

6. Overall, how confident do you feel in choosing the appropriate content for the following parts of your article?: *

Mark only one oval per row.

	No confidence	Low confidence	Moderate confidence	High confidence	Very high confidence
The “cover letter”					
The “Abstract"					
The “Title”					
The “Introduction"					
The “Methods" in general					
The “Ethical considerations					
The “Results" in general					
The “Tables" in the results					
The “Figures" in the results					
The “References"					
The “Acknowledgement" section					

7. Overall, how confident do you feel in choosing the appropriate content for the following parts of your Discussion?: *

Mark only one oval per row.

	No confidence	Low confidence	Moderate confidence	High confidence	Very high confidence
“Summarising” the main findings	◯	◯	◯	◯	◯
The “Limitations” of your work	◯	◯	◯	◯	◯
The “Strengths” of your work	◯	◯	◯	◯	◯
The relation of your work to others related works	◯	◯	◯	◯	◯
The “Implications for research”	◯	◯	◯	◯	◯
The “Conclusion"	◯	◯	◯	◯	◯

8. How confident do you feel in:

Mark only one oval per row.

	No confidence	Low confidence	Moderate confidence	High confidence	Very high confidence
Adhering to checklists such as CONSORT for your article?	◯	◯	◯	◯	◯

Domain 2

“Confidence” in the use of appropriate academic English language required for publishing articles in peer-reviewed medical journals.

9. Overall, how “confident” do you feel in appropriate use of:

Mark only one oval per row.

	No confidence	Low confidence	Moderate confidence	high confidence	Very high confident
“Active” versus passive voice?					
“Active” verbs versus the verb “to be”?					
“Semicolons”?					
“Short sentences” versus long sentences?					
“Simple words” versus complex words?					
“Abbreviations”?					
“Gerunds”?					
Past versus present versus future “Tenses”?					
“That” versus “Which”?					
“Dose” versus “Dosage”?					
“Sex” versus “Gender”?					
“Conjunctions”?					
Fewer versus more words					
Adjectives and adverbs					
“While” versus “although”					

10. Overall, how “confident” do you feel in appropriate:

Mark only one oval per row.

	No confidence	Low confidence	Moderate confidence	high confidence	Very high confident
Positioning of “Subordinate clauses”?	◯	◯	◯	◯	◯
Division into “Paragraphs”?	◯	◯	◯	◯	◯
Selection of “Word choices”?	◯	◯	◯	◯	◯

This content is neither created nor endorsed by Google.

Google Forms
